# Poor Cycling Performance of Rechargeable Lithium–Oxygen Batteries under Lean‐Electrolyte and High‐Areal‐Capacity Conditions: Role of Carbon Electrode Decomposition

**DOI:** 10.1002/advs.202300896

**Published:** 2023-06-20

**Authors:** Manai Ono, Jittraporn Saengkaew, Shoichi Matsuda

**Affiliations:** ^1^ Center for Green Research on Energy and Environmental Materials National Institute for Material Science 1‐1 Namiki Tsukuba Ibaraki 305‐0044 Japan; ^2^ NIMS‐SoftBank Advanced Technologies Development Center National Institute for Materials Science 1‐1 Namiki Tsukuba Ibaraki 305‐0044 Japan

**Keywords:** carbon nanotube, lean electrolyte, mass balance, metal–air batteries

## Abstract

There is growing demand for practical implementation of lithium–oxygen batteries (LOBs) due to their superior potential for achieving higher energy density than that of conventional lithium‐ion batteries. Although recent studies demonstrate the stable operation of 500 Wh kg^−1^‐class LOBs, their cycle life remains fancy. For further improving the cycle performance of LOBs, the complicated chemical degradation mechanism in LOBs must be elucidated. In particular, the quantitative contribution of each cell component to degradation phenomenon in LOBs under lean‐electrolyte and high‐areal‐capacity conditions should be clarified. In the present study, the mass balance of the positive‐electrode reaction in a LOB under lean‐electrolyte and high‐areal‐capacity conditions is quantitatively evaluated. The results reveal carbon electrode decomposition to be the critical factor that prevents the prolonged cycling of the LOB. Notably, the carbon electrode decomposition occur during charging at voltages higher than 3.8 V through the electrochemical decomposition of solid‐state side products. The findings of this study highlight the significance of improving the stability of the carbon electrode and/or forming Li_2_O_2_, which can decompose at voltages lower than 3.8 V, to realize high‐energy‐density LOBs with long cycle life.

## Graphical abstract

The complicated chemical/electrochemical reaction in the positive electrode of lithium–oxygen batteries under lean‐electrolyte and high‐areal‐capacity conditions is comprehensively analyzed using several in situ and ex situ techniques. This study demonstrates that the decomposition of carbon electrodes, which occurs associated with the electrochemical decomposition of solid‐state side products, is a critical factor that prevented prolonged cycling.

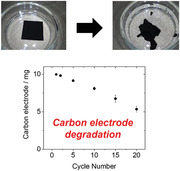

## 1 Introduction

There is growing societal demand for energy storage devices with superior high energy density. Although lithium‐ion batteries (LIBs) are extensively used as energy storage devices, their energy density is approaching the theoretical limit. Thus, the development of next‐generation rechargeable batteries. Lithium–oxygen batteries (LOBs) are specifically attracting significant attention owing to their high theoretical energy density.^[^ ^1^, ^2^, ^3^, ^4^, ^5^, ^6^ ^]^ However, most investigations on LOBs have been performed under excess‐electrolyte and low‐areal‐capacity conditions, resulting in their cell‐level energy density is lower than that of conventional LIBs.^[^ ^5^, ^6^ ^]^ Recently, a 500 Wh kg^−1^‐class LOB exhibiting stable discharge/charge cycling behavior at room temperature was demonstrated, which was achieved by minimizing the amount of the electrolyte as well as using novel electrode and electrolyte materials.^[^ ^7^, ^8^, ^9^, ^10^ ^]^ However, the 500 Wh kg^−1^‐class LOB showed stable cycling behavior for < 10 cycles. Thus, the complicated chemical degradation mechanism in LOBs must be elucidated, in order to further improve their cycle performance.

**Figure 1:**
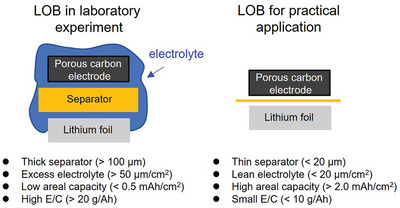
Schematic illustration of LOB.

The limited reaction efficiencies of the oxygen‐positive electrode and lithium‐negative electrode are thought to be principally involved in the degradation mechanism of LOBs.^[^ ^1^, ^2^, ^3^, ^4^, ^11^, ^12^ ^]^ Recent studies on the lithium negative electrode have revealed its unique degradation mechanism in lean‐electrolyte systems, which involves phenomena such as electrode expansion, electrolyte depletion, and chemical crossover from the positive electrode.^[^ ^13^, ^14^, ^15^, ^16^ ^]^ In contrast, the detailed mechanism for the oxygen‐positive electrode in lean‐electrolyte systems remains unclear. The degradation mechanism is believed to involve events such as decomposition of the electrolyte and redox mediator, oxidation of the carbon electrode, and pore‐clogging in the carbon electrode due to the accumulation of solid‐state side products.^[^ ^17^, ^18^, ^19^, ^20^, ^21^, ^22^, ^23^, ^24^, ^25^, ^26^ ^]^ However, such knowledge was obtained using LOBs with excess electrolytes (> 50 µL cm^−^ ^2^) and/or limited areal capacity conditions (< 1 mA cm^−^^2^) (**Figure** **1**). Thus, the quantitative contribution of each cell component to degradation phenomenon in LOBs under lean‐electrolyte and high‐areal‐capacity conditions remains unknown.

Based on these research backgrounds, in the present study, the complicated chemical/electrochemical reaction in the positive electrode of LOBs under lean‐electrolyte and high‐areal‐capacity conditions was comprehensively analyzed using several in situ and ex situ techniques. Using a solid‐state ceramic‐based separator to protect the lithium‐metal negative electrode, the side reaction with the negative electrode was considerably suppressed, which helped exclusively assess the degradation phenomena occurring in the oxygen‐positive electrode. The results indicated that carbon electrode decomposition was particularly detrimental to the prolonged cycling of LOBs.

## 2 Results and Discussion

In our experiments, stacked‐type LOB cells were utilized (Figure S1, Supporting Information). A self‐standing binder‐free single‐walled carbon nanotube (CNT) membrane with mass loading of 8.8 mg (2.2 mg cm^−^ ^2^) and 100‐µm‐thick lithium foil was used as the positive and negative electrodes, respectively. A solution of 0.5 mol L^−1^ LiTFSI + 0.5 mol L^−1^ LiNO_3_ + 0.2 mol L^−1^ LiBr in tetraethylene glycol dimethyl ether (TEGDME) was used as the electrolyte. A ceramic‐based solid‐state separator was used to protect the lithium‐metal negative electrode and minimize its undesired influence. Here, the ceramic‐based solid‐state separator was sandwiched between polyolefin layers and the same electrolyte was used on the positive and negative sides of the cell. Discharge/charge performance tests were conducted at a current density of 0.2 mA cm^−^ ^2^ capacity limitation of 2.0 mAh cm^−^ ^2^ and cutoff voltages of 2.0–4.5 V. The amount of the electrolyte in the LOB on the positive‐electrode side was controlled at 12.5 mg cm^−^ ^2^ Consequently, the ratio of the amount of the electrolyte to the areal capacity (E/C) was 6.25 g A^−1^h^−1^, which was sufficiently low for realizing cell‐level high‐energy‐density LOB.

**Figure 2:**
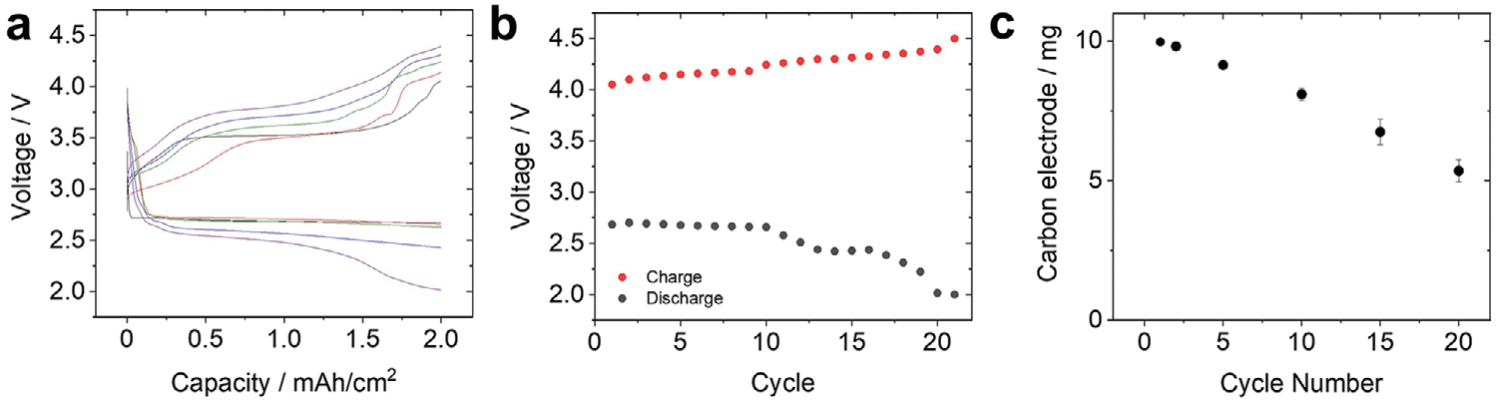
a) Discharge/charge profile of LOB at selected cycle (black curve: 1st, red curve: 5th, green curve: 10th, blue curve: 15th, purple curve: 20th). b) The final voltage during discharge (black circle) and charge process (red circle) was plotted against cycle number. c) The weight of carbon electrodes that were taken out from LOB cell at selected cycle was plotted against cycle number.

**Figure** **2**a shows representative voltage profiles of the LOB cell. During the discharge process, the cell exhibited a voltage plateau at ≈ 2.6 V. In charging, there can be seen the stable voltage plateau appeared at ≈ 3.5–3.6 V during middle part of the process. The voltage gradually increased up to 4.0 V at the end of the charging. The final voltage in discharge and charge process for each cycle was plotted against the cycle number (Figure 2b). Associated with the progress of discharge/charge cycle, the overpotential during both discharging and charging increased. As a result, the discharge voltage reached the cutoff voltage of 2.0 V at the 20th cycle. In a Li/Li symmetric cell fabricated with the same electrolyte composition, repeated cycling was achieved for over 30 cycles with an overpotential of < 100 mV (Figure S2, Supporting Information). Thus, the increase in the overpotential of the LOB was considered to be originated in the positive‐electrode reaction.

**Figure 3:**
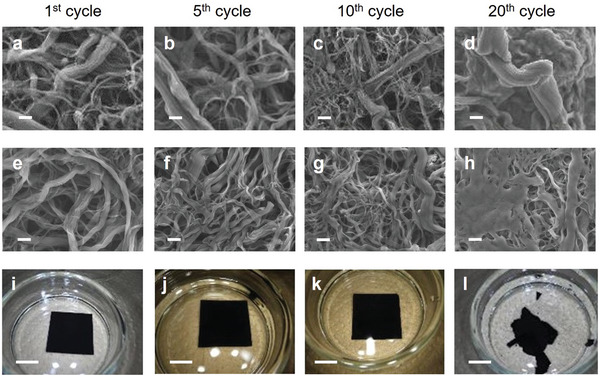
a–h) SEM and i–l) photographic images of carbon electrodes that were taken out from LOB cell at selected cycle. (a–d) The electrodes were washed by TEGDME and acetonitrile. (b–l) The electrodes were washed by TEGDME, acetonitrile, and water. Scale bars are (a–h)1 µm and (b–l) 1 cm.

One possible explanation for the increase in overpotential during repeated discharge/charge cycling is the accumulation of solid‐state side products on the carbon electrode, such as Li_2_CO_3_, which decreases the effective electrochemical surface area of the electrode. To investigate this possibility, the carbon electrodes that were taken out from the LOB cell after the 20th cycle were analyzed by scanning electron microscopy (SEM). For this experiment, the electrode removed from the LOB cell was, first, washed by TEMDME. After that washed with acetonitrile and then dried in vacuum. The SEM image of the electrode indicated that no apparent solid‐state products accumulated on the surface of the electrode (**Figure** **3**a–d; Figure S3, Supporting Information). X‐ray photoelectron spectroscopic (XPS). analysis also indicated that limited amounts of Li_2_CO_3_ accumulated on the electrode (Figure S4, Supporting Information). These results clearly revealed that the increase in overpotential during repeated discharging/charging originated from factors other than the accumulation of solid‐state side products on the carbon electrode.

**Figure 4:**
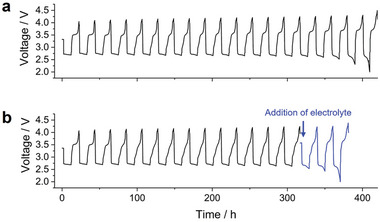
Voltage profile of LOB cell during repeated discharge/charge cycles. a) standard LOB cell, b) cycle was stopped at 15th cycle and electrolyte was added to carbon electrode, and then re‐start cycle test.

We investigated the weight change of the carbon electrode during discharge/charge cycling. The LOB cell was terminated at specific durations, and the carbon electrodes were taken out. The electrodes were washed by water to completely remove the solid‐state side products accumulated in the electrode, which was confirmed by XPS analysis (Figure S5, Supporting Information). In Figure 2c, the weight of the CNT electrode was plotted against the cycle number. There can be seen that the weight of the carbon electrode was found to linearly decrease with progress of cycle. Notably, the weight of the CNT electrode at the 20th cycle reached 5.2 mg (1.3 mg cm^−^ ^2^), which was 60% of the initial weight. SEM analysis of the CNT electrodes (Figure 3e–h) revealed that the morphologies of the CNT electrodes that were removed from the LOB cell after the 1st, 5th, and 10th cycle was almost identical to that of the pristine CNT electrode (Figure S3, Supporting Information). In contrast, the SEM image of the CNT electrode removed after the 20th cycle revealed aggregated structures. Photographic images of the CNT electrodes (Figure 3i–l) revealed that the CNT electrodes remained self‐standing up to the 10th cycle but collapsed structurally and cannot sustain their self‐standing nature after the 20th cycle (Figure 3l).

Next, the amounts of the TEGDME solvent and redox mediator species (LiNO_3_ and LiBr) in electrolyte were quantified. The solution taken out from positive electrode side in LOB cell was subjected to liquid chromatography–mass spectrometry (LC–MS) and ion chromatography (IC) analyses. In Figure S6 (Supporting Information), the weight of each component was plotted against the cycle number. The amount of TEGDME decreased with progress of discharge/charge cycle. As a result, the amount of the electrolyte remaining after the 20th cycle was ≈ 24 mg (6.0 mg cm^−^ ^2^), which was almost half the initial amount of TEGDME. It should be noted that the amount of electrolyte extracted from the non‐cycled cell is 47 mg (11.7 mg cm^−^ ^2^), which corresponds with 94% of the initially injected amount of electrolyte 50 mg (12.5 mg cm^−^ ^2^). The results also revealed that the amounts of the redox mediator species decreased with progress of cycle (Figure S6, Supporting Information). In our previous study, we experimentally confirmed the decomposition of anion (NO₃^−^ and Br^−^) proceeds during the operation of LOBs.^[^ ^27^ ^]^ Even in the present study, the decomposition of anion occurs, resulting in a decrease in the number of anions with the progress of repeated discharge/charge cycles.

Overall, the results obtained by analyzing the LOB cell after the 20th cycle, in which the discharge voltage reached the cutoff voltage condition, highlighted the following aspects: (i) limited accumulation of solid‐state side products in the carbon electrode, (ii) decrease in weight of the carbon electrode, and (iii) decrease in weight of the electrolyte (both solvent and redox mediator species). Therefore, the bottleneck for the prolonged cycling of LOBs had to be subsequently determined, with a focus on identifying the factor that decreased the discharge voltage. To that end, the effects of adding the electrolyte to the LOB cell when the discharge voltage reached the cutoff condition were investigated. Here, the amount of the added electrolyte was set to be equivalent to that of the decomposed electrolyte. Consequently, the amount of the electrolyte in the LOB cell after the electrolyte addition was identical to that in the initial cell. **Figure** **4** shows voltage profiles of the LOB cell before and after the electrolyte addition. The discharge voltage initially decreased to the cutoff voltage even after the addition of the electrolyte, suggesting that the shortage of the electrolyte did not primarily impede the prolonged cycling of the LOB cell. Thus, the insufficiency of the carbon electrode was presumably the crucial factor that increased the overpotential during repeated discharging/charging.

**Figure 5:**
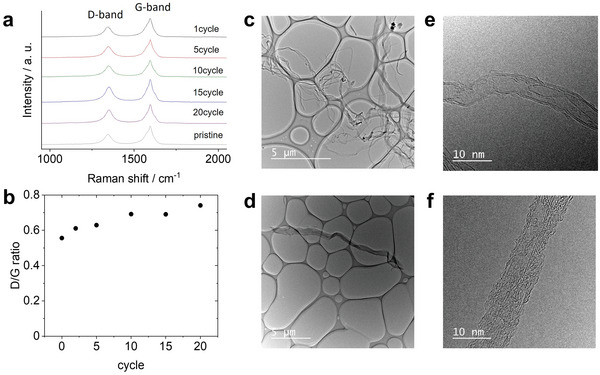
a) Raman spectroscopic analysis of carbon electrodes that were taken out from LOB cell at selected cycle. b) The value of Id against Ig was plotted against cycle number. c,e) TEM images of pristine CNT electrode and d,f) that were taken out from LOB cell after 20th cycle. Scale bar is (c,d) 5 µm and (e,f) 10 nm.

To get deep insight into the carbon electrode degradation phenomenon, we performed Raman analysis and transmission electron microscopy (TEM) analysis of the CNT electrodes that were removed from the LOB cell after different cycles. In the Raman spectra of the CNT electrode subjected to various cycles (**Figure** **5**a), peaks appeared at 1300 and 1600 cm^−1^, which were assigned to the D‐band and G‐band, respectively. The *I*_D_/*I*_G_ intensity ratio is typically used as an indicator of the defect density in CNTs.^[^ ^28^, ^29^ ^]^ A plot of *I*_D_/*I*_G_ against the cycle number (Figure 5b) indicated that *I*_D_/*I*_G_ gradually increased with increasing cycle number, suggesting an increase in the defect density and/or decrease in the CNT length. Figure 5c–f shows TEM images of the pristine CNT electrode (Figure 5c–e) and the CNT electrode removed from the LOB cell after the 20th cycle. The length of the CNTs in the pristine specimen is known to be 100–600 µm. This was confirmed by the low‐magnification TEM image of the pristine CNT sample. The high‐magnification TEM image revealed the single‐walled structure of the CNTs, which is consistent with previously reported results.^[^ ^28^, ^29^, ^30^ ^]^ However, the CNTs in the cycled specimen were < 20 µm long (Figure 5d). Additionally, the presence of defect structures was confirmed by high‐resolution TEM (Figure 5f). Therefore, the results of the Raman spectroscopy and TEM analyses clearly suggest the decomposition of the CNT electrode during repeated discharging/charging of the LOBs.

To further clarify the mechanism of the reaction in the positive electrode, on‐line MS analysis of the generated gas‐phase compounds, such as O_2_, CO_2_, and H_2_O, was performed. Figure S7 (Supporting Information) shows voltage profiles and time evolutions of the gas compounds generated during the 1st, 5th, 10th, and 20th charging cycles. In all cases, the yield of oxygen generated during charging was ≈ 80%. The oxygen generation occurred during the entire charging process in the 1st cycle. At the final stage of the charging process, oxygen generation abruptly decreased at a capacity of 1.9 mAh cm^−^^2^. CO_2_ generation was initiated at a capacity of 1.7 mAh cm^−^ ^2^ and increased rapidly toward the end of the charging. As a result, the total yield of oxygen generated during the 1st charging was 115 µmol, which corresponded to 82% of the theoretical value. This is essentially consistent with previous results obtained using similar cell setups.^[^ ^31^, ^32^ ^]^ The yield of CO_2_ generated was 27.6 µmol. It should be noted that even after the charging is completed with capacity limitation of 2.0 mA cm^−^^2^, the generation of O_2_ and CO_2_ does not quickly decrease, instead gradually decreases. We considered that it takes certain time for generated gas to completely get out from the inside of pores in CNT electrode. The oxygen generated during the 5th cycle started to decrease at a capacity of 1.7 mAh cm^−^^2^, and its total yield was 112 µmol. However, the yield of CO_2_ was 43 µmol, which was considerably higher than that of the 1st charging cycle. Gas‐generation profiles similar to those of the 5th charging cycle were obtained for the 10th and 20th cycles. The yields of the generated gases are summarized in **Table** **1**. Ex situ weight measurements of the carbon electrode indicated that ≈ 0.025 mg ( = 27 µmol) of carbon decomposed during each cycle. Thus, the results of in situ MS analysis clearly revealed that more than half of the CO_2_ generated during the charging originated from the carbon electrode decomposition.

**Table 1:** Amount of generated O_2_ and CO_2_ during charging process

Cycle	O_2_ µmol^−1^	CO_2_ µmol^−1^
1	115.41	27.68
5	112.95	43.13
10	104.63	45.37
15	103.85	52.68
20	105.06	49.63
Charge start	<1	1.68

The results of in situ MS experiments clearly revealed that most of the CO_2_ was generated at voltages higher than 3.8 V. Thus, we hypothesized that the carbon‐electrode‐decomposition reaction could have occurred predominantly at voltages higher than 3.8 V. To confirm this hypothesis, experiments were performed using a LOB with a charging cutoff voltage of 3.8 V (Figure S8, Supporting Information). Although the stable voltage profiles were acquired up to the 10th cycle, the overpotential for the discharge process started to increase at the 15th cycle. As a result, the discharge voltage reached the cutoff voltage at the 20th discharge cycle. We also performed the in situ MS experiment of LOB cell with cutoff voltage of 3.8 V, revealing the limited amount of CO_2_ generation in this condition (Figures S9 and S10, Supporting Information). In addition, ex situ experiment of the electrode taken out from LOB cell after 20th cycle revealed that the solid‐state products in the electrode were uniformly distributed on the electrode surface (Figure S11a, Supporting Information), suggesting the amount of accumulated Li_2_CO_3_‐like side products keep increasing with progress of cycle. Figure S11b (Supporting Information) showed the SEM image of the CNT electrode that was taken out from LOB cell with cutoff voltage of 3.8 V, after washing with water to completely remove the solid‐state side products accumulated in the electrode. Photographic images of the CNT electrodes revealed that the CNT electrodes remained self‐standing condition even after 20th cycle (Figure S12, Supporting Information). These results clearly revealed that the structure of CNT electrode remains even after 20th cycle, which is sharp contrast with the case of cutoff voltage of 4.5 V.

We also investigated the cutoff voltage dependency of carbon electrode degradation (Figure S13, Supporting Information). First, there can be seen that ≈ 40% of carbon electrode is decomposed after 20th cycle when cutoff voltage is 4.2 or 4.5 V. In such case, the CNT electrode does not remain their self‐standing nature. In contrast, in case with cutoff voltage with 3.8 V, the decomposition of carbon electrode is suppressed to 20%. In this case, CNT electrode sustains their self‐standing property even after 20th cycle. These results clearly revealed that major part of carbon electrode decomposition proceeds at the voltage region over 3.8 V. However, we also mentioned that certain amount of carbon decomposition reaction also proceeds at the voltage region < 3.8 V. We also performed Raman spectroscopic analysis of CNT electrode that taken out after repeated discharge/charge cycle with cutoff voltage of 3.8 V condition. The results revealed that the increase of *I*_D_/*I*_G_ value keep increasing with progress of cycle even in case with cutoff voltage of 3.8 V condition (Figure S14, Supporting Information), although the increase rate is smaller than the case with cutoff voltage of 4.5 V condition.

Finally, the detailed mechanism of the CNT‐electrode‐decomposition reaction had to be clarified. One possible candidate is the direct electrochemical decomposition of the CNT electrode. To investigate the contribution of the electrochemical oxidation of the CNT electrode during charging, the fabricated LOB cell was charged but not discharged. The acquired voltage and gas‐generation profiles (Figure S15, Supporting Information) indicated that the voltage promptly increased and reached the cutoff voltage of 4.5 V at a capacity of 1 mAh cm^−^ ^2^ Moreover, CO_2_ generation—which was limited—was initiated at a voltage of 3.8 V. Because the amount of CO_2_ generated was significantly lower than that in the 1st charging cycle, these results revealed that the CO_2_ generation during charging did not occur via simple electrochemical oxidation.

The other possible mechanism of carbon electrode decomposition during charging at voltages higher than 3.8 V is the decomposition of CNT electrodes associated with the electrochemical decomposition of Li_2_O_2_ or Li_2_CO_3_.^[^ ^33^ ^]^

(1) $${\mathrm{Li}}_{2}O_{2}+C\to {\mathrm{CO}}_{2}+2Li^{+}+2e^{-}$$

(2) $$2Li_{2}{\mathrm{CO}}_{3}+C\to 3CO_{2}+4Li^{+}+4e^{-}$$

The Li_2_O_2_ that did not decompose at voltages lower than 3.8 V, and the Li_2_CO_3_ generated via the chemical reaction between Li_2_O_2_ and the carbon electrode or TEGDME, were considered to decompose electrochemically at voltages higher than 3.8 V. Consequently, the carbon electrode containing these solid‐state products also decomposed owing to the electrochemical decomposition of these products, forming CO_2_ at voltages higher than 3.8 V. Such degradation of CNT electrode leads the increase of over‐potential during repeated discharge/charge process. For the detailed mechanism, following four issues should be considered. (i) decrease of surface area of CNT electrode due to the decrease of the mass of CNT, (ii) decrease of surface area of CNT electrode due to the decrease of electric conduction through CNT electrode, (iii) increase of IR drop due to the decrease of electric conduction through CNT electrode. In addition, the decrease of micro‐sized pore in CNT electrode results in inefficient oxygen transport through CNT electrode. These factors result in the increase of over‐potential during repeated cycle of LOBs.

## 3 Conclusion

The degradation mechanism of LOBs under lean‐electrolyte and high‐areal‐capacity conditions was investigated. The conclusions of a series of analytical investigations conducted in this study are outlined henceforth. (i) Although the electrolyte degradation intensified with increasing repeated discharge/charge cycling, the scarcity of the electrolyte minimally influenced the cycle life of the LOBs. (ii) Most of the Li_2_CO_3_‐like solid‐state side products decomposed during charging at voltages higher than 3.8 V. (iii) Severe decomposition of the carbon electrode occurred during cycling, and an equivalent amount of CO_2_ was generated during charging at voltages higher than 3.8 V. Because the carbon electrode used in these experiments merely decomposed via simple electrochemical oxidation at voltages up to 4.5 V, the carbon electrode was considered to deteriorate through the decomposition of its solid‐state side products. The findings of this study are anticipated to guide future investigations on the advancement of LOBs. In particular, a detailed understanding of the reaction at the interface between the carbon electrode and solid‐state products is crucial for realizing practical high‐energy–density LOBs with long cycle life.

## 4 Experimental Section

### Materials

Tetraethylene glycol dimethyl ether: Tetraglym (battery grade) and lithium bis(trifluoro methanesulfonyl)imide : LiTFSI (Li battery grade) were purchased from Kishida Chemical Co., Ltd. and used as received. Lithium nitrate (LiNO_3_, 99.99% trace metals basis) and lithium bromide (LiBr, 99.995% trace metals basis) were obtained from Sigma–Aldrich Co., LLC and were dried under vacuo at 120 °C for > 3 days. Liquid electrolytes, 0.5 mol L^−1^ LiTFSI + 0.5 mol L^−1^ LiNO_3_ + 0.2 mol L^−1^ LiBr/TEGDME were prepared in a dry condition. Their water contents were confirmed < 100 ppm by the Nittoseiko Karl Fischer Moisture Meter CA‐31. A lithium foil (100 µm thick) was obtained from The Honjo Chemical Corp. and cut into 20 mm^2^. The CNT (carbon nano fiber unwoven cloth) sheet was obtained from Zeon Corporation (100 µm thick, 2 mg cm^−^ ^2^) and cut into 20 mm^2^ and was used after dried in vacuo at 110 °C for 15 h. The carbon paper TORAYCA H‐030 was purchased from Toray Industries, Inc. and cut into 20×22 mm then dried in vacuo at 110 °C for 15 h. A Li conducting solid electrolyte (LICGC‐AG‐01, 180 µm thick) was obtained from Ohara, Inc. and cut into 25 mm^2^. Porous polyethylene membrane separators (20 or 5 µm thick) obtained from Hosen, and Toray Industries, Inc., respectively, were cut into 22 mm^2^ and kept in the drying condition for > 1 week before use. All dried cell components were stored in a dry booth its dew point lowered to −50 °C for more than overnight before use.

### Cell Assembly and Discharge/Charge Cycling Tests

An original test cell (stainless steel, inner diameter: 45 mm, depth: 15 mm) equipped with a gas inlet and outlet was assembled in a dry room. A Li metal electrode (100 µm thick, 20 mm square) was put onto a 1 mm thickness of the stainless spacer as a current corrector, a porous polyethylene membrane (20 µm thick, 22 mmsquare) infiltrate a 10 µL of electrolyte, a Li^+^ conducting solid electrolyte, a polyethylene membrane (5 µm thick, 22 mm square) with another 10 µL of electrolyte, an above‐mentioned electrolyte impregnated CNT electrode, a carbon sheet as a gas diffusion layer, and a 1 mm thickness of stainless spacer with springs to fasten at 60 kPa were stacked in the cell. The dead volume of the cell was ≈ 24.5 mL, for excess amount of O_2_ inside space. Here, a solid electrolyte was used for separating cathode and anode, in order to avoid poisoning electrodes with decomposition and/or redox shuttling on the Li surface, and two types of porous polyethylene membranes were for preventing direct contact with electrodes and solid electrolyte. The sealed cell was purged with dry O_2_ flow at 20 ml min^−1^. The discharge/charge tests were carried out by the HOKUTO DENKO HJ1020mSD8 at 0.2 mA cm^−2^ for 10 h at ambient temperature followed by the resting time for > 30 min at each interval between discharge and charge.

### Analysis of Gas Phase Components

For on‐line MS analysis, the generated gases were directly to the MS detector by the Canon Anelva Quadrupole Mass Spectrometer M‐401GA‐DM equipped with a capillary tube (internal diameter: 0.05 mm, length: 7 m). After discharge, the test cell was purged with excess He (50mLmin^−1^) for 1 min to remove the remaining O_2_. He as a carrier gas was flowed at 5 ml min^−1^ and keep it for 2 h before charge. The former on‐line measurement was carried out at 100 µV applied voltage, the m/z range from 11 to 110 at ambient condition.

### Analysis of Liquid Phase Components

Liquid chromatography for the organic component, and ion chromatography for the ions including inorganic materials. After the above measurement, the loss amount of the liquid electrolytes in cathode were extracted with water and analyzed by the Acquity H‐class Ultra High‐Pressure Liquid Chromatography (UPLC, Xevo G2‐S QTof, Waters) system coupled with a mass spectrometer and Ion chromatography (ICS‐2100, Dionex). The carbon paper, cathode electrode, and cathode separator were immersed in ultrapure water and sonicated for 10 min, then filtrated. The obtained cathode samples were diluted properly for each measurement. The liquid TEGDME volume was measured by LC‐MS by extracting the electrolyte from the positive electrode in the cell after the measurements.

### Analysis of Carbon Electrodes

The electrochemically cycled cathode electrode was compared with the pristine one by weight loss measurement, morphological observation, chemical composition analysis, and structural investigation of CNTs. The samples for weighing were obtained by washing with water to remove electrolyte and solid‐state products in cycled cathodes. The cathode electrodes after the discharging/charging cycles were immersed in excess ultrapure water at 40 °C for > 6 h and wiping surplus water, and this process was repeated several times. The electrodes were dried in vacuum and put into the Ar‐filled glovebox at leased overnight before measurements. The surface morphological measurement of the positive electrode was carried out using a field emission scanning electron microscope (JSM‐7800F, JEOL) equipped with an energy‐dispersive X‐ray spectrometer (X‐MaxN 50, Oxford), and the surface chemical species were analyzed using X‐ray photoelectron spectra using a VersaProbe II Scanning XPS Microprobe (ULVAC‐PHY). The samples after the electrochemical measurements were cut and utilized from the weight measurement samples described above. Additionally, in order to analyze the solid‐state products, SEM and XPS measurements were also performed using the samples washed as follows. The cell was disassembled in glove box after electrochemical measurements. The carbon electrodes were first washed by TEMDME. After that electrodes were immersed in excess dry acetonitrile at 40 °C for > 8 h and wipe surplus solvent, and this process was repeated several times. The carbon electrode with remaining solid products was obtained after dried in the glovebox. Sample preparations for SEM and XPS were performed in a glove box and measured using an unexposed chamber or vessel, respectively. The structural defects in CNTs were investigated using microscopic Raman spectrometer (Raman Touch‐vis–NIR, Nanophoton) with a 532 nm laser, and the defects and the length of CNTs were observed using a Transmission Electron Microscope (JEM‐ARM200F, JEOL). The samples for Raman spectroscopy were used the same samples as the weight measurement described above. The TEM samples as dispersed solution was prepared using pristine or electrochemically cycled carbon electrodes of the weight measurement described above. The samples were obtained by sonication with a piece of electrode into 1‐propanol for > 4 h. Then dispersion was dropped on a grid and air‐dried before measurement.

## Conflict of Interest

The authors declare no conflict of interest.

## Supporting information

Supporting Information

## Data Availability

The data that support the findings of this study are available from the corresponding author upon reasonable request.
