# Supplementary material for: Poor Cycling Performance of Rechargeable Lithium–Oxygen Batteries under Lean‐Electrolyte and High‐Areal‐Capacity Conditions: Role of Carbon Electrode Decomposition
Source: Adv Sci (Weinh). 2023 Jun 20;10(24):2300896. doi: 10.1002/advs.202300896 (PMC10460881; doi:10.1002/advs.202300896)
Supplement: Supplementary file 1 — Supporting Information [file ADVS-10-2300896-s001.pdf]

Supporting Information

for *Adv. Sci.*, DOI 10.1002/advs.202300896

Poor Cycling Performance of Rechargeable Lithium–Oxygen Batteries under Lean-Electrolyte and High-Areal-Capacity Conditions: Role of Carbon Electrode Decomposition

*Manai Ono, Jittraporn Saengkaew and Shoichi Matsuda**

<u>**Supporting Information**</u>

Poor Cycling Performance of Rechargeable Lithium–Oxygen Batteries under Lean-Electrolyte and High-Areal-Capacity Conditions: Role of Carbon Electrode Decomposition

Manai Ono[1], *Jittraporn Saengkaew*[1], *Shoichi Matsuda* [1,2 *]

[1]*Center for Green Research on Energy and Environmental Materials, National Institute for Material Science, 1-1 Namiki, Tsukuba, Ibaraki 305-0044, Japan*

[2]*NIMS-SoftBank Advanced Technologies Development Center, National Institute for Materials Science, 1-1 Namiki, Tsukuba, Ibaraki 305-0044, Japan*

Corresponding Author

E-mail: MATSUDA.Shoichi@nims.go.jp

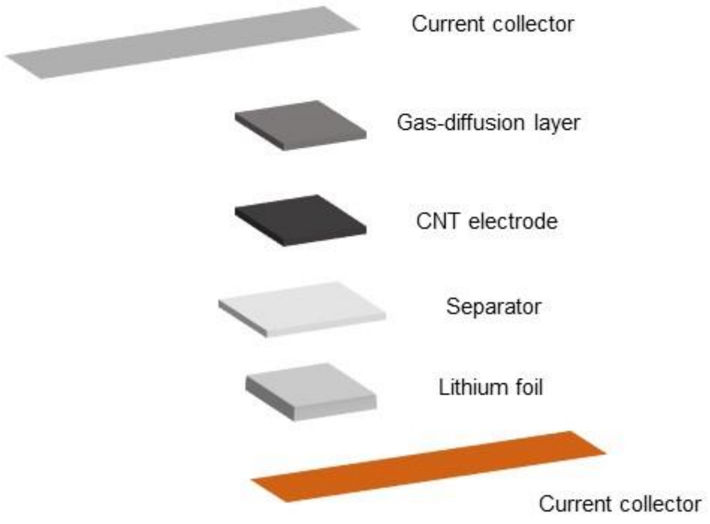

Figure S1. Schematic illustration of stacked type cell configuration of LOB used in the present study.

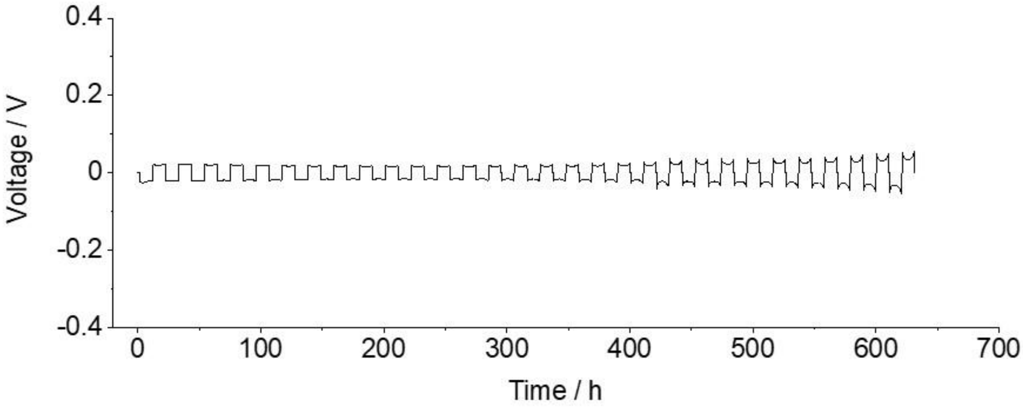

Figure S2. Voltage profile of repeated lithium deposition/dissolution reaction in Li/Li symmetric cell with current density of 0.4 mA/cm^2 and capacity limitation of 0.4 mA/cm^2.

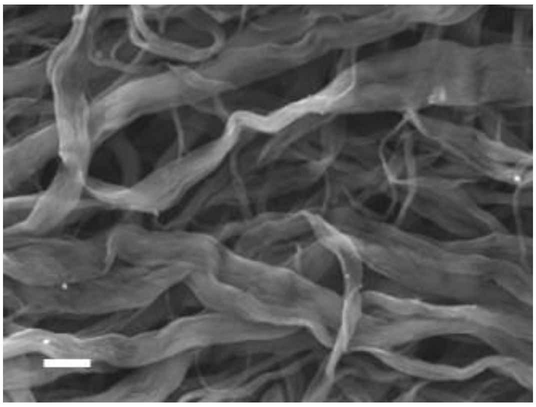

Figure S3. SEM image of pristine CNT electrode. Scale bars are 1 μm.

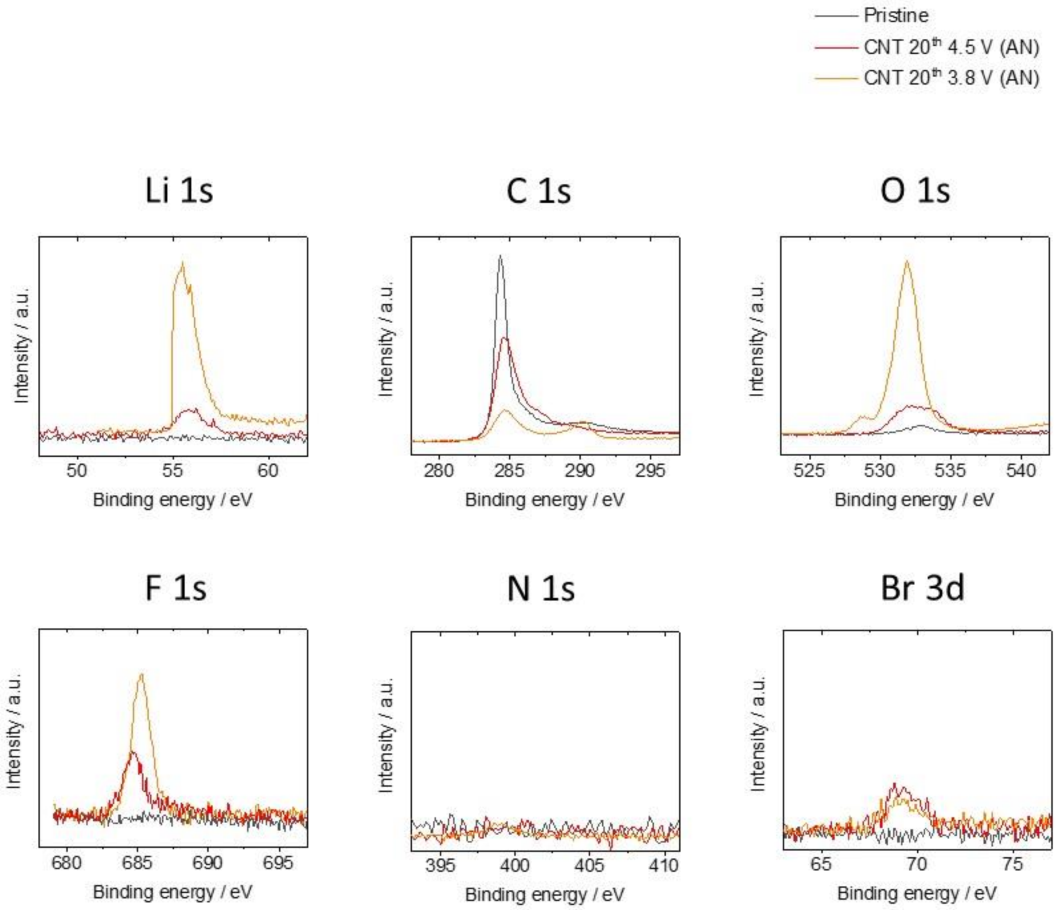

Figure S4. XPS analysis of carbon electrodes that were taken out from LOB cell at selected cycle. The electrodes were washed by acetonitrile.

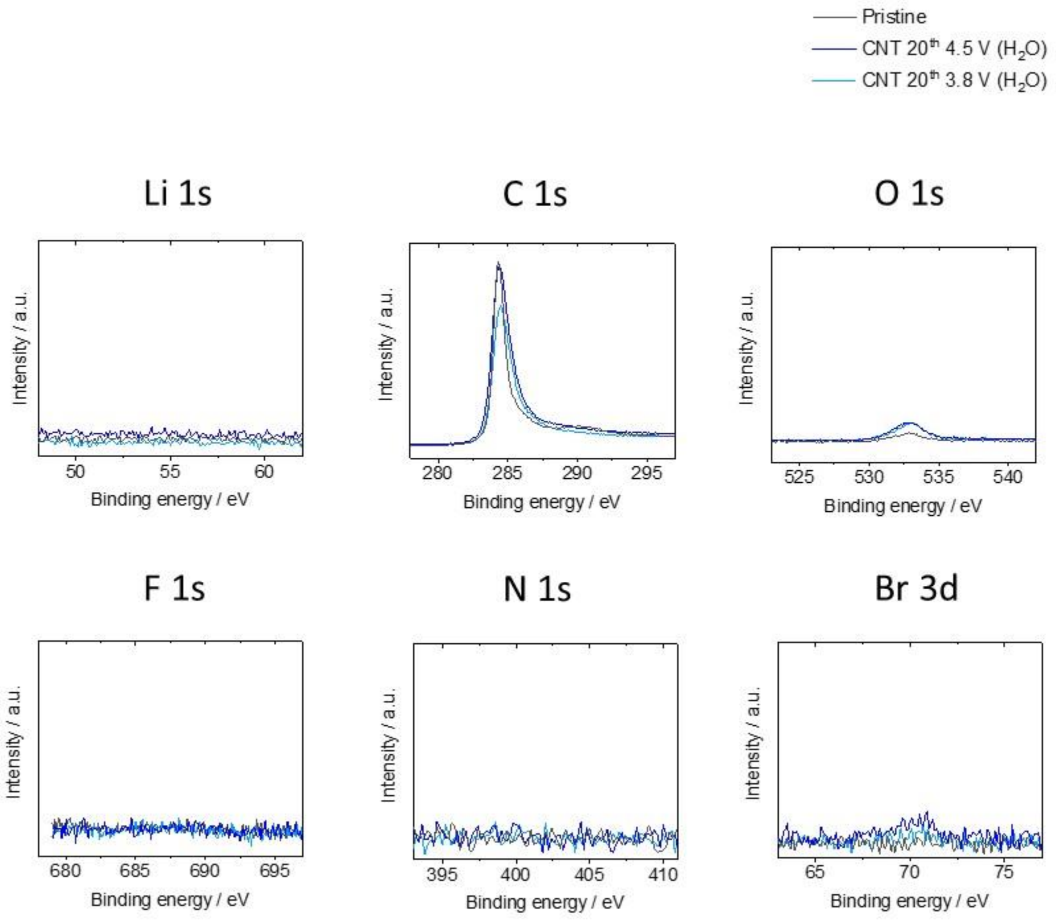

Figure S5. XPS analysis of carbon electrodes that were taken out from LOB cell at selected cycle. The electrodes were washed by acetonitrile and water.

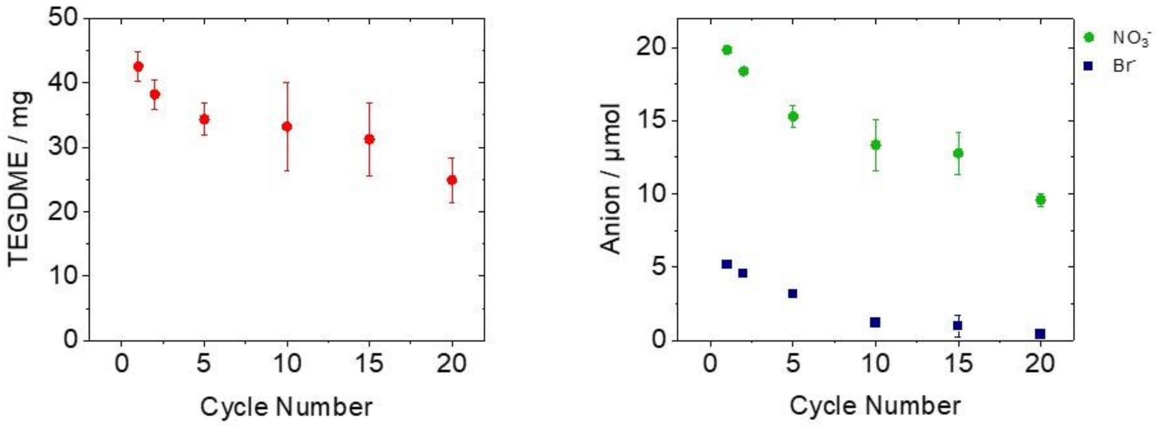

Figure S6. The amount of TEGDME solvent, NO_3^- and Br^- that were taken out from carbon electrode at selected cycle were plotted against cycle number.

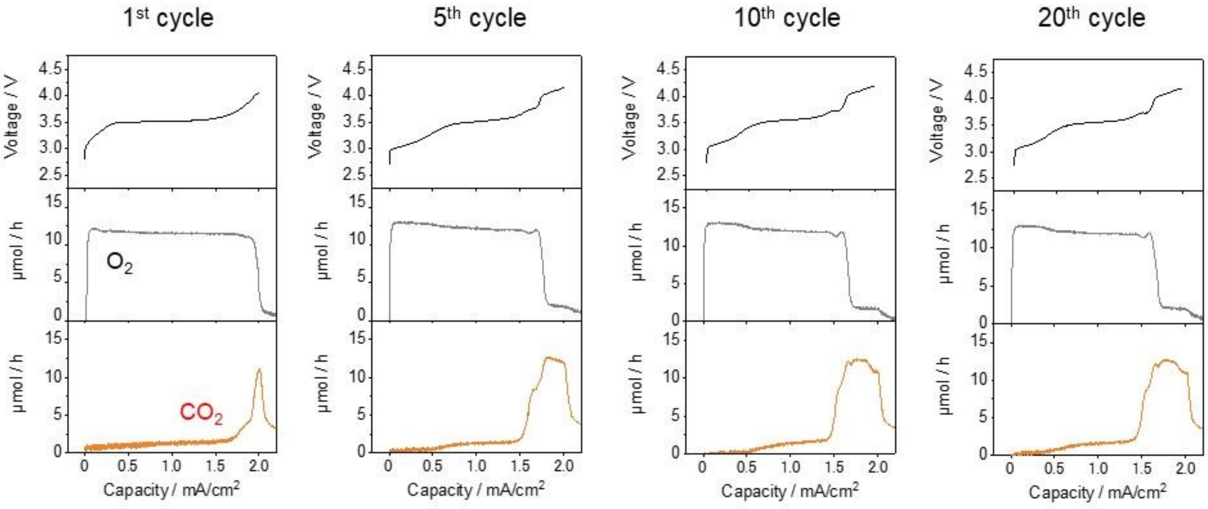

Figure S7. In situ MS analysis of LOB cell at 1st, 5th, 10th, and 20th charging process.

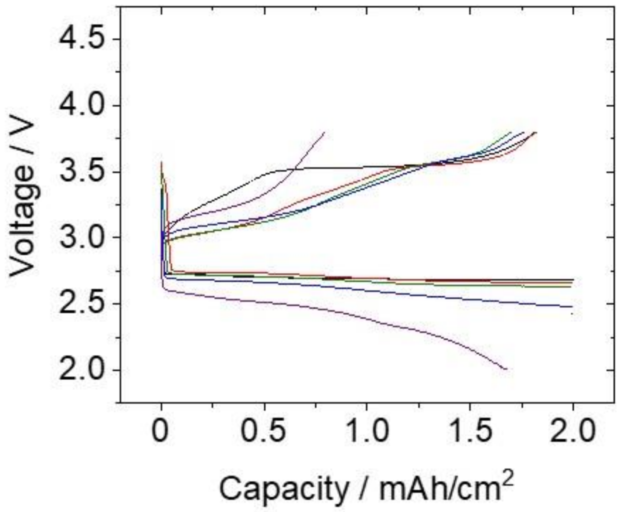

Figure S8. (a) Discharge /charge profile of LOB at selected cycle with cutoff voltage of 3.8 V (black curve: 1st, red curve: 5th, green curve: 10th, blue curve: 15th, purple curve: 20th).

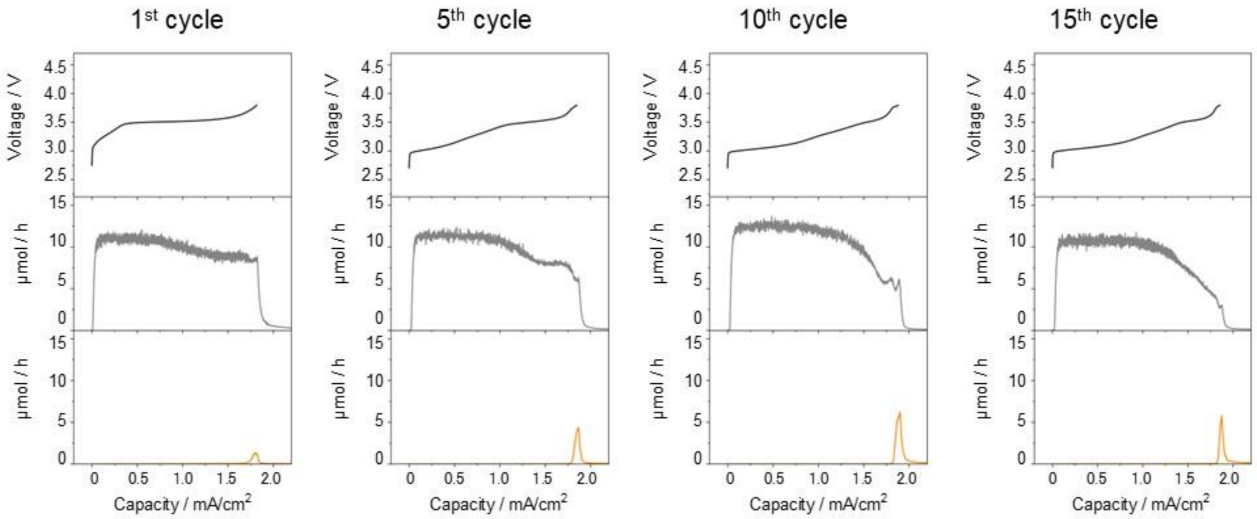

Figure S9. In situ MS analysis of LOB cell at 1st, 5th, 10th, and 15th charging process with cutoff voltage of 3.8 V condition.

Cycle	O_2 / μmol	CO_2 / μmol
1	92.63	0.96
5	92.85	1.69
10	101.73	2.88
15	85.16	2.19

Figure S10. Amount of generated O_2 and CO_2 during charging process with cutoff volage of 3.8 V condition.

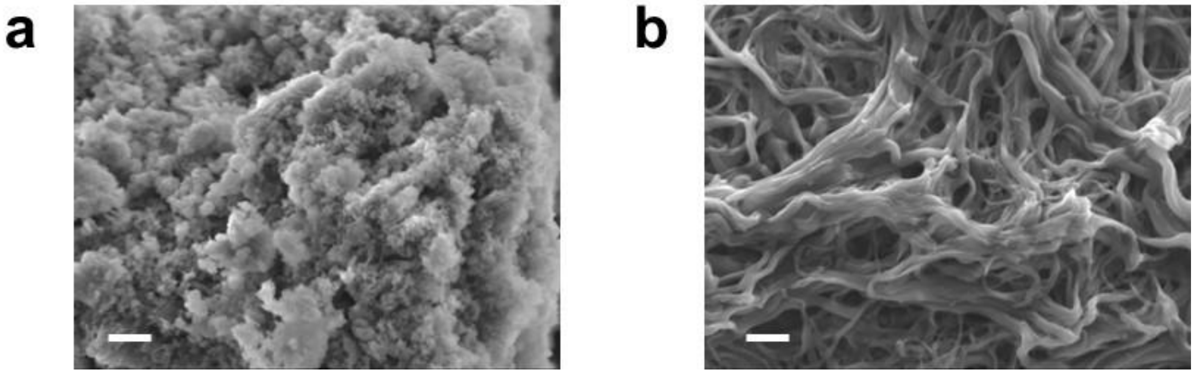

Figure S11. SEM images of carbon electrodes that were taken out from LOB cell after 20th cycle with cutoff voltage of 3.8 V. (a) The electrodes were washed by acetonitrile. (b) The electrodes were washed by acetonitrile and water. Scale bars are 1 μm.

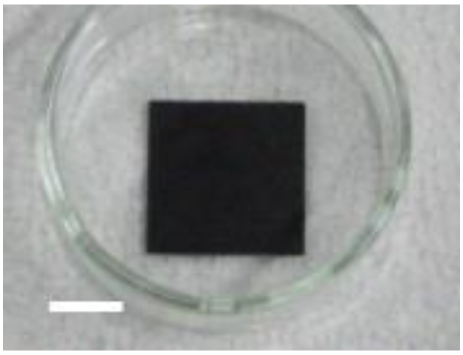

Figure S12. Photographic image of carbon electrodes that were taken out from LOB cell after 20th cycle with cutoff volage of 3.8 V condition. Scale bar is 1cm.

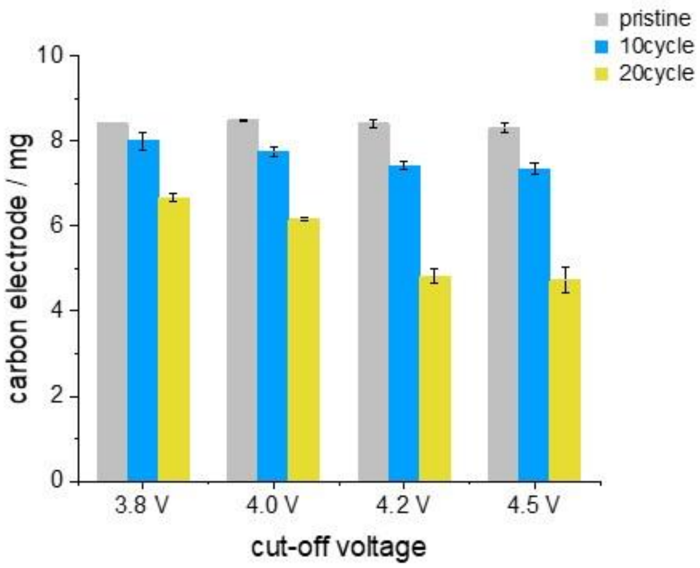

Figure S13. The wight of carbon electrodes that were taken out from LOB cells after 20th cycle with cutoff voltage of 3.8, 4.0, 4.2 and 4.5 V.

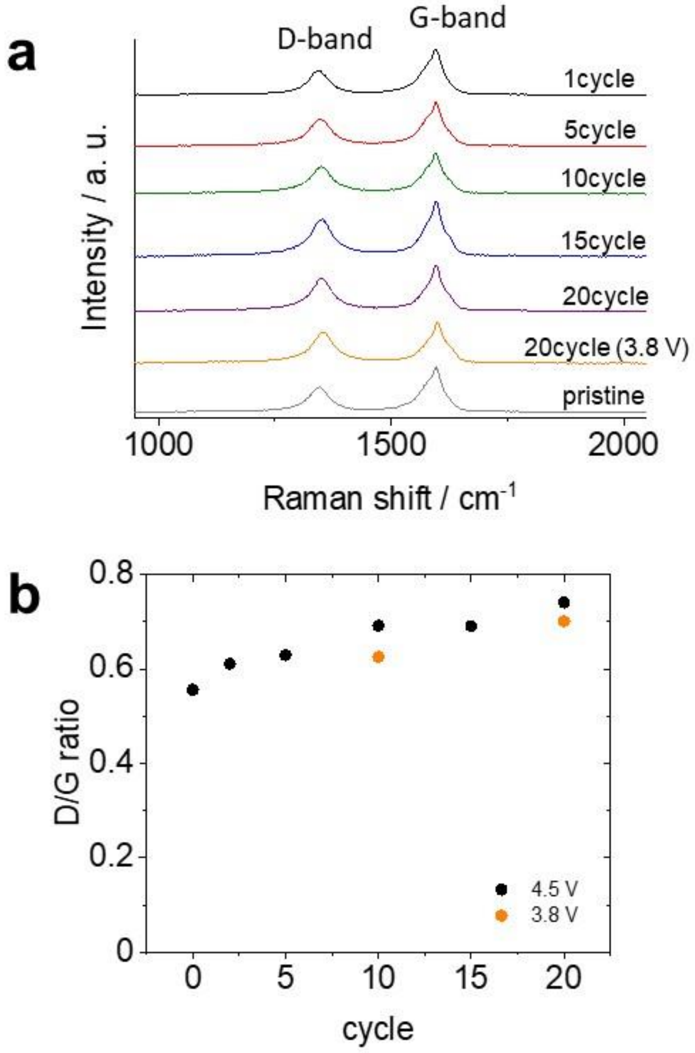

Figure S14. (a) Raman spectroscopic analysis of carbon electrodes that were taken out from LOB cell after 20th cycle with cutoff volage of 3.8 V condition. (b) Value of Id against Ig was plotted against cycle number.

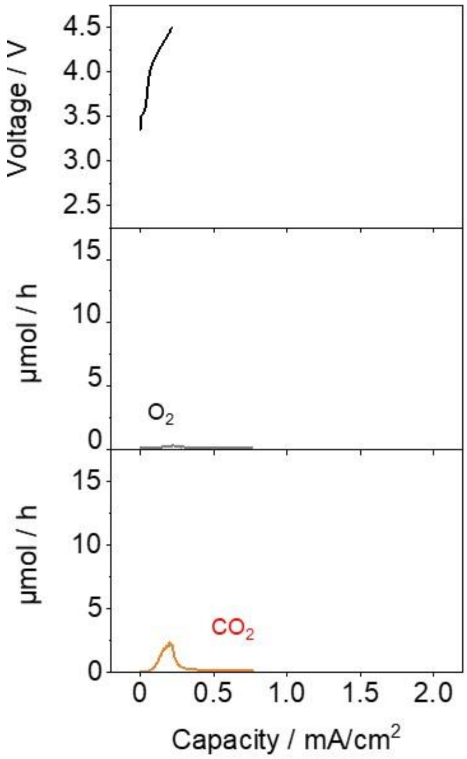

Figure S15. In situ MS analysis of LOB cell. The cell was subjected to 1st charging process without 1st discharging process.
